# Editorial: Bacterial metabolomics approach towards antimicrobials and resistance

**DOI:** 10.3389/fmicb.2023.1222594

**Published:** 2023-05-30

**Authors:** Vijay Soni, Zhe Wang, Vinayak Singh

**Affiliations:** ^1^Division of Infectious Diseases, Weill Department of Medicine, Weill Cornell Medicine, New York, NY, United States; ^2^Shanghai Key Laboratory of Veterinary Biotechnology, School of Agriculture and Biology, Shanghai Jiao Tong University, Shanghai, China; ^3^Shanghai Collaborative Innovation Center of Agri-Seeds, School of Agriculture and Biology, Shanghai Jiao Tong University, Shanghai, China; ^4^Holistic Drug Discovery and Development Centre (H3D), University of Cape Town, Rondebosch, South Africa; ^5^South African Medical Research Council Drug Discovery and Development Research Unit, Institute of Infectious Disease and Molecular Medicine, University of Cape Town, Rondebosch, South Africa

**Keywords:** antimicrobial resistance (AMR), drug mechanism of action, LC-MS, metabolomic biomarker, microbial metabolomics, novel drug candidates, pathogenic bacteria antimicrobial assay

The rising prevalence of bacterial infections and antimicrobial resistance (AMR) are becoming a growing global concern. According to the United Nations Environment Programme, by 2050 AMR will be responsible for almost 10 million deaths every year and if not controlled it will cost around USD 3.4 trillion annually. Therefore, there is an urgent need to develop novel approaches and treatments. Metabolomics presents a promising solution by accelerating the discovery of microbial metabolic changes during infection and upon antibiotic treatment (Figure 1). Thus, enabling researchers to identify potential novel drug targets and strategies to develop better therapeutic interventions to combat AMR. In this Research Topic, we have compiled several interesting articles covering novel discoveries around this theme.

**Figure 1:**
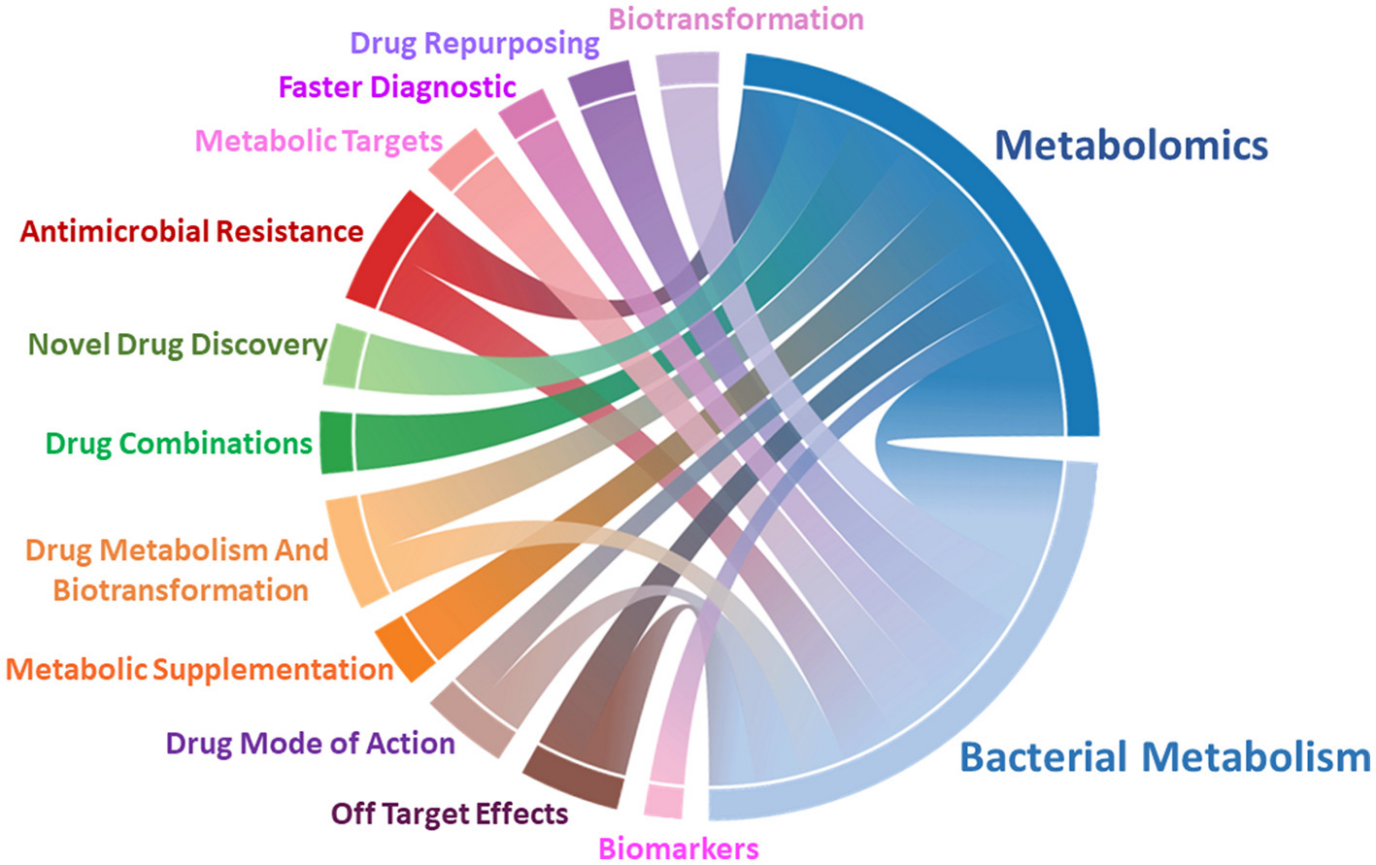
Metabolomics plays a central role in studying bacterial metabolism. Chord diagram representing correlations of metabolomics, microbial metabolism, and their application in drug development, diagnostic, and to understand mechanism of drug resistance and action.

Wu et al. evaluated bacterial metabolomics of *Staphylococcus aureus* upon Berberine hydrochloride (BBR) treatment and found significant inhibition of cell wall biosynthesis, perturbance in shikimate metabolism and elevated amino sugar production, nucleic acid biosynthesis and nucleotide sugar metabolism. Two stress resistance-related biomarkers, pyridine dicarboxylic acids, were downregulated while oxidized phospholipid levels were increased.

In the same direction, Chen et al. reported the antimicrobial activity of essential oil (EO), isolated from fresh mature *Litsea cubeba*, against *Cutibacterium acnes*. Metabolomics upon EO treatment showed alteration of 86 metabolites and 34 of them belonged to cell wall pathways, amino acid biosynthesis, energy production, and carbohydrate metabolism. ATP levels were extremely low and the activities of glycolytic enzymes and the Wood-Werkman cycle (malate dehydrogenase, pyruvate kinase, and pyruvate carboxylase activities were decreased, and hexokinase activity was increased) were perturbed significantly upon EO treatment.

Further, Zhu et al. investigated the synergistic behavior of polymyxin-B, meropenem, and sulbactam against multi-drug resistant *Acinetobacter baumannii*. A short (15 min) treatment combination of all three antibiotics substantially affected the levels of metabolites

involved in the cell wall, cell outer membrane, peptidoglycan, fatty acid, lipopolysaccharide, glycerophospholipid, and nucleotide metabolism. Interestingly, a longer (1 h) treatment of the double and triple combinations significantly interrupted the amino acids and nucleotide biosynthesis and central carbon metabolism.

Zhou et al. reported an interesting example of discovering a metabolite-based antibiotic adjuvant by comparing the metabolic profiles of drug-sensitive (SCH-S) and gentamicin-resistant *Salmonella* Choleraesuis (SCH-R). Non-targeted metabolomics revealed extensive disruption in energy and central carbon metabolism along with the dysregulations of amino acid biosynthesis, nucleotide production, vitamin and cofactor metabolism, and fatty acid biogenesis. Remarkably, D-ribose was the most suppressed metabolite in SCH-R and enhanced the efficacy of gentamicin against SCH-R and clinical multidrug-resistant strains. They suggested that D-ribose activates pentose phosphate pathway, glycolysis, and tricarboxylic acid cycle which induced the NADH levels, polarizes the electron transport chain, and raises the proton motive force to increase drug uptake, and kills the bacteria.

Study by Zhang et al. revealed the application of matrine-chitosan hydrogels (MCH) to cure subclinical mastitis, a bacterial infection-mediated inflammation in bovine mammary glands. Using metabolomics and 16S sequencing they investigated the biological response to MCH in mastitis-affected cows. They discovered that relative abundances of bacterial communities such as Staphylococcus, Aerococcus, and Corynebacterium_1 were reduced after 7 days of MCH treatment. MCH infusion was also found to be associated with differentially expressed metabolites in milk, such as fatty acyls, flavonoids, sphingolipids, and glycerophospholipids. This study provides new understandings about the immunoregulatory mechanisms to matrine and its role in controlling mastitis in dairy cows.

Moreover, Singh et al. compared different methods of metabolite extraction that included freeze-thaw cycle (FTC) and sonication cycle (SC) individually and in combination (FTC+SC). Using ESI-LC-MS/MS, they found that both FTC and SC methods were efficient in isolating AMR-associated metabolites from *S. aureus*, while combining these methods does not improve the efficiency. Basically, each method showed some level of selectivity toward metabolites and the selection of the right method should be based on the possible class and type of metabolites.

Mohammadi et al. developed Microbial Containment Device (MCD), a novel high-throughput 96-well sampling system, to simplify boundary flux analysis and captured complex microbial metabolic phenotypes. Using a semi-permeable membrane, MCD allowed diffusion of water-soluble metabolites from bacterial culture reservoir into a bacteria-free analytical well. MCD required minimal sample handling and enabled single-step metabolomics to identify different bacterial strains and their responses to antibiotics. This can be very useful for large-scale metabolomics studies as it is compatible with traditional commercial autosamplers of LC-MS systems.

Nutrients and growth conditions play a crucial role in metabolism, especially in detecting AMR. Zeng et al. developed Salmonella-Shigella (SS) ceftazidime/avibactam (CZA)-selective medium for clinical screening of CZA-resistant carbapenem-resistant Enterobacterales (CRE) isolates from pure culture and simulated clinical polymicrobial specimens such as stool samples. With 100% specificity and sensitivity, the screening efficiency of SS-CZA media remained natural for different resistance determinants that were found in tested isolates.

Consecutively, Mapar et al. reported how commercial media such as Mueller Hintonmedium can affect the *in vitro* metabolic phenotypes and formulated Biomarker Enrichment Medium (BEM)—a complex defined media derived from Roswell Park Memorial Institute (RPMI) medium) as an alternative for metabolomic assays. Combining substrate exclusion, isotope-labeling, and nutritional supplementation methods they have identified specific nutrients required for unique biomarkers production by pathogenic bacteria. Using this information, they have proposed BEM as an alternative to complex undefined media for metabolomics experiments, clinical diagnostics, and drug discovery, where stable metabolic profiles play a crucial role.

A review from Xu et al. summarized the importance of nitrogen metabolism for Mycobacteria and showed the role of related genes and pathways in bacterial survival, virulence, and suggested a direction to develop novel targeted small molecular antimicrobials. Authors audited extensive literature to elaborate on the role of nitrogen metabolazing genes such as *glnA1, pknG, groES, groEL2, ansP2, ansA, Rv322c, argB*, and *argF*. Further, they have described the way forward, gaps, and recommendations to target nitrogen metabolism using specific inhibitors to control the emergence of AMR mycobacteria.

Finally, we want to express our gratitude to all contributing authors and reviewers who made it possible. We believe that this collection would assist readers to comprehend novel aspects of metabolomics and its applications in understanding the bacterial metabolism and controlling AMR.

## Author contributions

VSo conceptualized, collected all information, and wrote the first draft. ZW participated in the writing of remaining sections. VSi and VSo edited the final draft. All authors contributed to the article and approved the submitted version.

